# Rates Of New ADRD Diagnosis Within 1 Year Of Entering Assisted Living Facilities: General Care Versus Memory Care

**DOI:** 10.1093/geroni/igaf122.3836

**Published:** 2025-12-31

**Authors:** Gauri Gadkari, Cassandra Hua, Jennifer Bunker, Kali Thomas, Eric Jutkowitz

**Affiliations:** Brown University, Providence, Rhode Island, United States; University of Massachusetts Lowell, Lowell, Massachusetts, United States; Johns Hopkins University, Baltimore, Maryland, United States; Johns Hopkins University, Baltimore, Maryland, United States; University of Minnesota, Minneapolis, Minnesota, United States

## Abstract

Assisted living (AL) is a growing option for long-term residential care among older adults. Some ALs hold specialized licenses to provide memory care (MC) and are better equipped to support residents experiencing cognitive decline, making them more likely to attract individuals anticipating such needs, regardless of their diagnosis status. Using Medicare claims, we identified beneficiaries who newly moved to ALs during 2020 and 2021 across 34 states with available MC licensure data. We examined rates of new Alzheimer’s disease and related dementias (ADRD) diagnoses within a year from entry into AL. Residents with an existing ADRD diagnosis (no MC: 24%, MC: 27%) were excluded, resulting in a final cohort of 95,207 beneficiaries. We fit logistic regression models to examine whether having MC licensure was associated with higher probability of new ADRD diagnoses. Risks were derived from predictive margins after adjusting for demographics, co-morbidities, and prior health utilization. The adjusted risks showed on average 10.9% (95% CI: 10.6-11.1) of beneficiaries receive an ADRD diagnosis within a year of moving to an AL without MC compared to 11.7% (95% CI: 11.3-12.0) among those who move to AL with MC. The small difference was statistically significant (p < 0.001). These findings suggest that while individuals in ALs with MC are more likely to receive an ADRD diagnosis, the difference in rate of new diagnosis is small. Many ALs providing MC also offer general care and thus may have a sufficient population not at risk for ADRD.

